# Base Damage within Single-Strand DNA Underlies *In Vivo* Hypermutability Induced by a Ubiquitous Environmental Agent

**DOI:** 10.1371/journal.pgen.1003149

**Published:** 2012-12-13

**Authors:** Kin Chan, Joan F. Sterling, Steven A. Roberts, Ashok S. Bhagwat, Michael A. Resnick, Dmitry A. Gordenin

**Affiliations:** 1 Chromosome Stability Section, Laboratory of Molecular Genetics, National Institute of Environmental Health Sciences, National Institutes of Health, Research Triangle Park, North Carolina, United States of America; 2 Department of Chemistry, Wayne State University, Detroit, Michigan, United States of America; Duke University, United States of America

## Abstract

Chromosomal DNA must be in single-strand form for important transactions such as replication, transcription, and recombination to occur. The single-strand DNA (ssDNA) is more prone to damage than double-strand DNA (dsDNA), due to greater exposure of chemically reactive moieties in the nitrogenous bases. Thus, there can be agents that damage regions of ssDNA *in vivo* while being inert toward dsDNA. To assess the potential hazard posed by such agents, we devised an ssDNA–specific mutagenesis reporter system in budding yeast. The reporter strains bear the *cdc13-1* temperature-sensitive mutation, such that shifting to 37°C results in telomere uncapping and ensuing 5′ to 3′ enzymatic resection. This exposes the reporter region, containing three closely-spaced reporter genes, as a long 3′ ssDNA overhang. We validated the ability of the system to detect mutagenic damage within ssDNA by expressing a modified human single-strand specific cytosine deaminase, APOBEC3G. APOBEC3G induced a high density of substitutions at cytosines in the ssDNA overhang strand, resulting in frequent, simultaneous inactivation of two reporter genes. We then examined the mutagenicity of sulfites, a class of reactive sulfur oxides to which humans are exposed frequently via respiration and food intake. Sulfites, at a concentration similar to that found in some foods, induced a high density of mutations, almost always as substitutions at cytosines in the ssDNA overhang strand, resulting in simultaneous inactivation of at least two reporter genes. Furthermore, sulfites formed a long-lived adducted 2′-deoxyuracil intermediate in DNA that was resistant to excision by uracil–DNA N-glycosylase. This intermediate was bypassed by error-prone translesion DNA synthesis, frequently involving Pol ζ, during repair synthesis. Our results suggest that sulfite-induced lesions in DNA can be particularly deleterious, since cells might not possess the means to repair or bypass such lesions accurately.

## Author Summary

A cell's genome is encoded within double-strand DNA. Yet DNA must exist transiently in single-strand form to template transcription, replication, and repair. As DNA is more prone to damage in single-strand (ssDNA) than double-strand (dsDNA) form, there can be agents that mutate ssDNA, but not dsDNA. Since existing experimental systems cannot identify mutagens specifically mutating ssDNA inside cells, we devised a system of yeast strains, containing three closely-spaced reporter genes, for this purpose. We exposed yeast, under conditions where reporter DNA is single-stranded, either to an enzyme (APOBEC3G) or to a chemical (sulfites) that modifies cytosine, resulting in clusters of mutations that inactivated multiple reporter genes. Neither agent induced mutations in control strains where reporter genes remained double-stranded, confirming that both are potent ssDNA–specific mutagens within cells. Finally, our approach ascertained molecular mechanisms of action by which agents can mutate ssDNA specifically within cells, an area that warrants much investigation following reports that ssDNA–specific damage accounts for similar mutation clusters, and up to 40% of all mutations, in various cancers.

## Introduction

The genetic information of cellular organisms is encoded within double-strand DNA (dsDNA) genomes. Yet during DNA transactions such as replication, transcription, or recombination, the DNA exists transiently in single-strand form. It has long been appreciated that moieties in the nitrogenous bases of DNA are susceptible to damage, from both endogenous and exogenous sources, and that the double helical duplex structure can protect such moieties from chemical attack, due to Watson-Crick base pairing as well as base stacking [1]. Conversely, single-strand DNA (ssDNA) is significantly more vulnerable to various forms of chemical modification. For example, spontaneous deamination of cytosine to uracil occurs at least 100-fold more rapidly in ssDNA than in dsDNA [1]. Similarly, the alkylation of either N1 in adenine or N3 in cytosine, each of which would result in lesions that block replication, occurs much more readily in ssDNA [2]. Spontaneous depurination and depyrimidination each occurs four-fold more rapidly in ssDNA than in dsDNA [3]. In addition, it has been shown that transcription of DNA is associated with increased frequency of mutation and recombination, possibly due in part to the increased susceptibility of transient ssDNA (of the non-coding strand) to endogenous DNA damage [4]. Finally, lesions formed in transient ssDNA during genome replication [5] likely would have to be bypassed by translesion synthesis (TLS), which can be error-prone, i.e. mutagenic [6], [7]. Thus, regions of ssDNA are expected to be at greater risk of damage than dsDNA *in vivo*, since there could be many agents that are not reactive against dsDNA, but are reactive enough to damage ssDNA.

Indeed, we showed previously that regions of ssDNA formed by resection at double-strand breaks (DSBs) [5], [6] or uncapped telomeres [6], [7], as well as ssDNA within dysfunctional replication forks [5], were prone to increased mutagenesis. Damage-induced mutagenesis in such ssDNA can lead to the formation of clusters of multiple simultaneous mutations, with a mutation density comparable to the expected density of induced DNA lesions. For instance, methylmethanesulfonate (MMS)-induced clusters of multiple point mutations originated from methylated bases within ssDNA that was formed by resection from a DSB or in transient ssDNA formed during replication [5]. The number of mutations within an individual cluster, generated as a single ssDNA-associated event, often exceeded the total number of mutations in the rest of the genome that were accumulated during approximately 25 generations of growth in the presence of MMS. Therefore, a mutagen that acts weakly on dsDNA is sufficiently reactive to induce mutation clusters in transiently formed ssDNA.

Strikingly, similar clusters of mutations were discovered in four types of malignant tumors [5], [8]. A considerable proportion of such mutation clusters clearly exhibited the signature of endogenous single-strand specific mutagens, namely APOBEC cytosine deaminases. Moreover, from 9% to 42% of all mutations in these tumors occurred at sequence motifs preferred by APOBEC enzymes, indicating that ssDNA-specific mutagenesis could be a significant contributor to carcinogenesis. It is therefore possible that other ssDNA-damaging agents, with properties that are not as well-understood as that of APOBEC enzymes, very well could contribute to mutagenesis also. Thus, it is crucial to identify such mutagens and to elucidate their mechanisms of action.

In order to make accurate assessments of the potential hazard posed by such ssDNA-damaging agents, we devised a subtelomeric triple reporter gene system in budding yeast as a facile means to identify and characterize ssDNA-specific mutagens. This reporter system takes advantage of the propensity of such mutagens to generate clusters of multiple point mutations spanning more than 10 kb, which can inactivate multiple closely-spaced reporter genes simultaneously. First, we validated the approach by expressing a modified human APOBEC3G in the reporter strains, resulting in clusters of mutations caused by deamination of cytosines in the ssDNA reporter region. We then used the reporter strains to characterize the mutagenicity of sulfites, a class of sulfur (IV) oxide compounds that is present in the environment (mainly due to combustion of fossil fuels [9]) and in the food supply (>0.34% by weight of some foods consists of sulfites [10]). Using a concentration of bisulfite (1%) that is similar to levels measured in foods, we confirmed that bisulfite is a very potent *in vivo* deaminating agent that reacted specifically with cytosines in ssDNA to generate large clusters of mutations. But unlike uracil formed by enzymatic cytosine deamination, the main mutagenic lesion caused by sulfites (5,6-dihydrouracil-6-sulfonate) was refractory to excision by uracil-DNA N-glycosylase, and often was bypassed with the aid of an error-prone TLS polymerase. The reaction of either APOBEC3G or bisulfite with ssDNA each resulted in a distinctive strand-coordinated, multi-mutation signature, reminiscent of mutation clusters found in cancers [5], [8]. Our results further underscore the necessity of identifying ssDNA-specific mutagens and investigating the molecular mechanisms by which they act *in vivo*.

## Results

### A reporter system to identify ssDNA–specific mutagens

**Figure 1:**
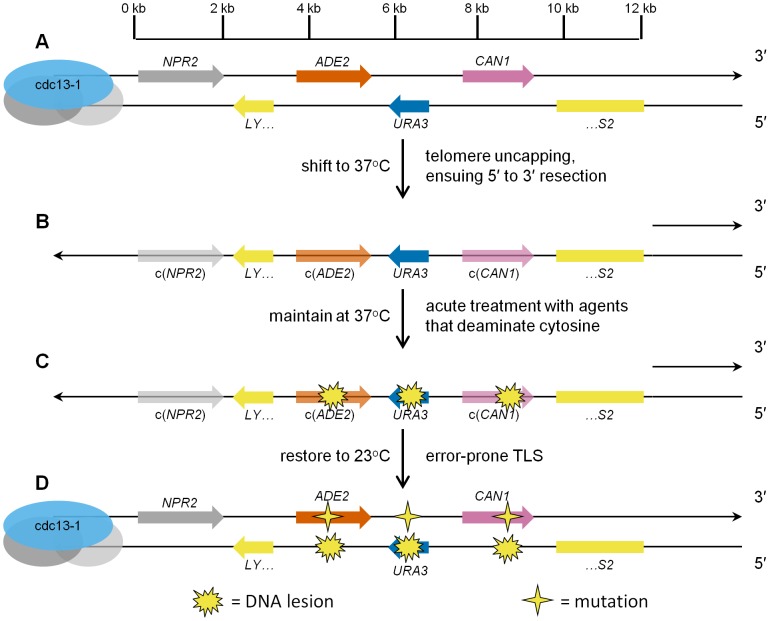
Schematic representation of ssDNA mutagenesis reporter system. (A) Three reporter genes, *ADE2*, *URA3*, and *CAN1*, were relocated from their respective native genomic loci into the subtelomeric region of Chromosome V, within a haploid budding yeast strain bearing the temperature-sensitive *cdc13-1* mutation, thus creating strain ySR127. The 0 kb mark in the scale bar denotes the start of unique DNA sequence (conversely, the end of telomeric repeat sequences). (B) Shifting ySR127 cells to 37°C results in telomere uncapping. Subsequent 5′ to 3′ resection results in a long 3′ ssDNA overhang. c(*ADE2*) and c(*CAN1*) denote the complement of the two genes. (C) Cells then undergo acute treatment with agents that deaminate cytosine, e.g. human APOBEC3G or sodium bisulfite, which induce lesions in the 3′ ssDNA overhang. (D) Shifting back to permissive temperature (23°C) restores the subtelomeric DNA to double-stranded form. Error-prone bypass of lesions formed in ssDNA generates a strand-coordinated, multi-mutation signature, which is detected by simultaneous loss of function in two or more of the reporter genes, and verified by sequencing of individual multi-loss-of-function isolates.

Since conventional reporter systems for detection of mutagenesis *in vivo* are not suited for the identification of mutagens that preferentially target ssDNA, we constructed a reporter system that is designed expressly for this purpose. We deleted three reporter genes, *ADE2*, *URA3*, and *CAN1*, from their native genomic locations, and re-inserted them into the left subtelomeric region of Chromosome V, near a *de novo* telomere, in haploid yeast (see Figure 1A). Due to the *cdc13-1* temperature-sensitive mutation [11], when these cells are shifted to restrictive temperature (e.g. 37°C), the protein complex that protects the telomeres dissociates, and ensuing 5′ to 3′ enzymatic resection [12] generates long 3′ ssDNA overhangs that can encompass the reporter gene region (see Figure 1B). The long ssDNA triggers the DNA damage checkpoint, arresting the cells in G_2_ [13]. With the subtelomeric reporter in an ssDNA state, we then treat cells with an agent of interest to determine if it reacts with the ssDNA to cause mutations (see Figure 1C). If lesions were induced in the ssDNA, it is likely that when the cells are returned to permissive temperature (23°C), and the subtelomeric DNA is restored to a double-strand state, translesion DNA synthesis involving specialized error-prone DNA polymerases could create mutations opposite the damaged bases (see Figure 1D).

This series of events would generate a characteristic, strand-coordinated, multi-mutation signature, which can be detected by plating cells on canavanine media with low adenine, selecting for *CAN1* loss of function (such colonies are resistant to canavanine, i.e. are Can^R^), and enabling facile screening for *ADE2* loss of function by red pigmentation. Also, we can assess whether *URA3* function remains intact by replica plating onto URA dropout media. In order to determine if the mutagenesis is ssDNA-specific, the frequency of mutagenesis in the subtelomeric reporter strain is compared to the mutation frequency in a negative control strain which has the same reporter in the middle of Chromosome II. The reporter region in the mid-chromosome control strain is located >300 kb from the nearest telomere, thus ensuring the reporter remains double-stranded throughout the mutagen exposure. In summary, this reporter system would detect any ssDNA-associated increase in mutation frequency quite readily. But just as importantly, the system is optimized for the discovery of agents that are strongly mutagenic toward ssDNA, as these agents would be expected to inactivate multiple reporter genes simultaneously, due to mutations originating from multiple lesions within the ssDNA overhang strand.

### The expression of human APOBEC3G induces localized hypermutability within subtelomeric ssDNA

In the current study with the triple-gene subtelomeric reporter system, we concentrated on agents that induce cytosine deamination, because this reaction is known to occur much more readily in ssDNA [1]. We started from a well-understood enzymatic agent directly creating uracil from cytosines in ssDNA to serve, in effect, as a positive control for the overall experimental approach, as well as a means to investigate the fate of uracil that is generated in ssDNA. As such, we tested whether expression of human APOBEC3G in our reporter strains does, indeed, induce a strand-coordinated, multi-mutation signature due to lesions in the subtelomeric ssDNA overhang. APOBEC3G is a member of a family of cytosine deaminases that function in innate and adaptive immunity within mammals [14]. APOBEC3G restricts retroviral infection by deaminating cytosines (to form uracils) in the minus strand DNA copy of the retroviral genome, thus resulting in hypermutated proviral genomes [15]. We cloned a modified version of human APOBEC3G [16], [17] into the pCM252 tetracycline-regulatable centromeric vector [18] to generate pCM252-A3G. We then transformed pCM252-A3G into WT and *ung1*Δ subtelomeric reporter strains, as well as the mid-chromosome negative control strain. *UNG1* encodes the sole uracil-DNA N-glycosylase in budding yeast, i.e. the enzyme that excises uracil from DNA to initiate base excision repair (BER) [19]. Reporter strains bearing pCM252-A3G were shifted to 37°C in the presence of 10 µg/mL doxycycline, thus inducing APOBEC3G expression as subtelomeric ssDNA was being formed.

**Figure 2:**
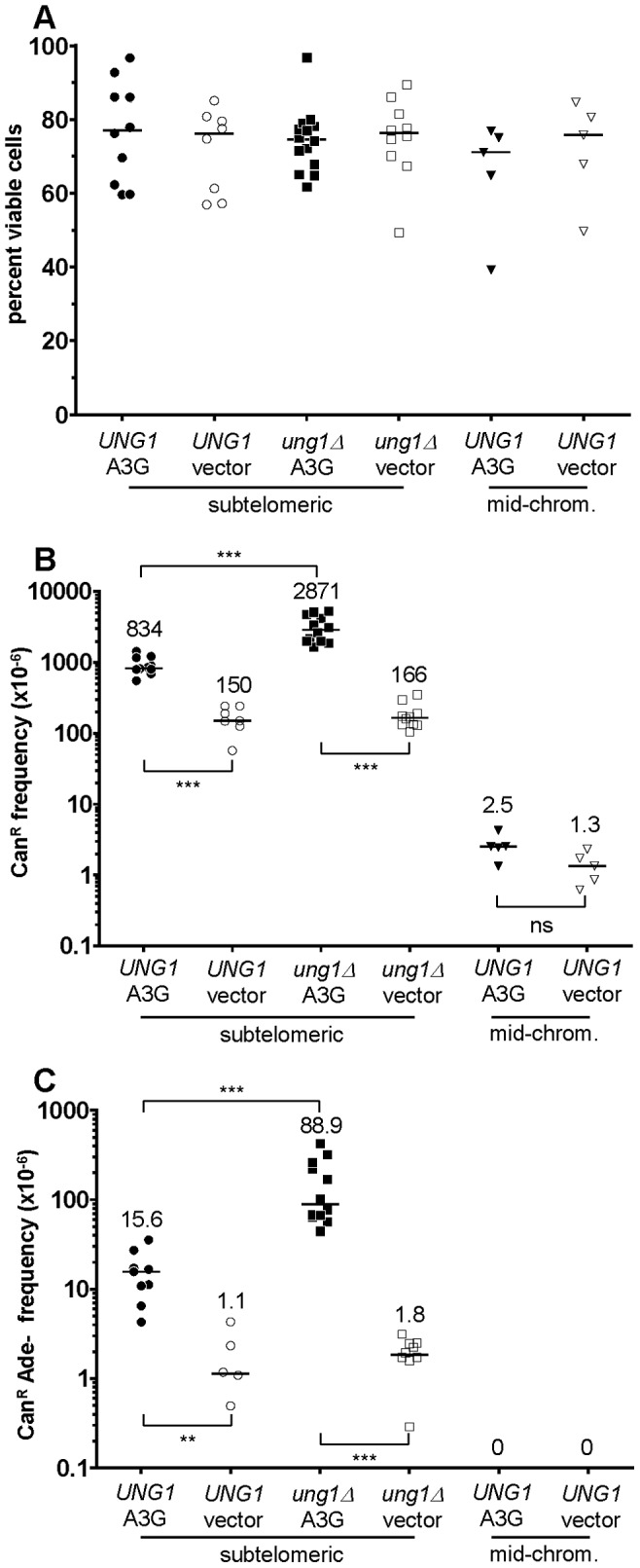
The activity of human APOBEC3G on the ssDNA mutagenesis reporter system. For all graphs: each data point represents the value from an independent experimental replicate; each bar represents the median value across all independent replicates of a given genotype and treatment combination; * denotes P<0.05; ** denotes P<0.01; *** denotes P<0.001; and ns denotes P>0.05. (A) Expression of human APOBEC3G from a tetracycline-regulatable plasmid was well-tolerated in reporter strains. Median viability was >70%. (B) APOBEC3G induced an increased frequency of *CAN1* loss of function, specifically in ssDNA. Mutagenicity was enhanced over three-fold in cells deleted for *UNG1*, which encodes uracil-DNA N-glycosylase. Notice the lack of mutagenesis in mid-chromosome reporter controls, where the DNA remained double-stranded. (C) Similarly, APOBEC3G induced an increased frequency of simultaneous *CAN1* and *ADE2* double loss of function, in an ssDNA-dependent manner. Deletion of *UNG1* enhanced mutagenicity by almost six-fold.

As shown in Figure 2A, APOBEC3G expression was well-tolerated in all reporter strains. APOBEC3G caused a significant increase in the frequency of *CAN1* inactivation in both WT and *ung1*Δ subtelomeric reporter strains over empty vector controls (P<0.001 in both cases, see Figure 2B), but not in the mid-chromosome negative control. Even more significantly, expression of APOBEC3G induced a 14-fold increase in the frequency of simultaneous loss of both *CAN1* and *ADE2* function in WT cells, and a 49-fold increase in *ung1*Δ cells (P<0.01 for WT, P<0.001 for *ung1*Δ, see Figure 2C). These results are consistent with the expectation that APOBEC3G would induce mutations in multiple reporter genes only when ssDNA is present. This is a form of localized hypermutability, since the mutation frequency within the ssDNA can be ∼1000-fold greater than that found in other locations within the genome, which can be assumed to exist as canonical dsDNA (see also [5], [7]). Curiously, the mutagenicity of APOBEC3G was over three-fold higher in the *ung1*Δ background (P<0.001 when comparing WT to *ung1*Δ), suggesting that even when repair by BER is not possible (since the would-be template strand had been removed by enzymatic resection), excision of uracils to generate abasic sites affords a considerably greater chance to avoid mutation than the alternative, i.e. not excising the uracils at all, which virtually guarantees resultant C to T transitions at each site of deamination.

### The spectrum of APOBEC3G-induced mutations confirms that multi-loss-of-function isolates arose from mutations at multiple cytosines in the subtelomeric ssDNA overhang

**Figure 3:**
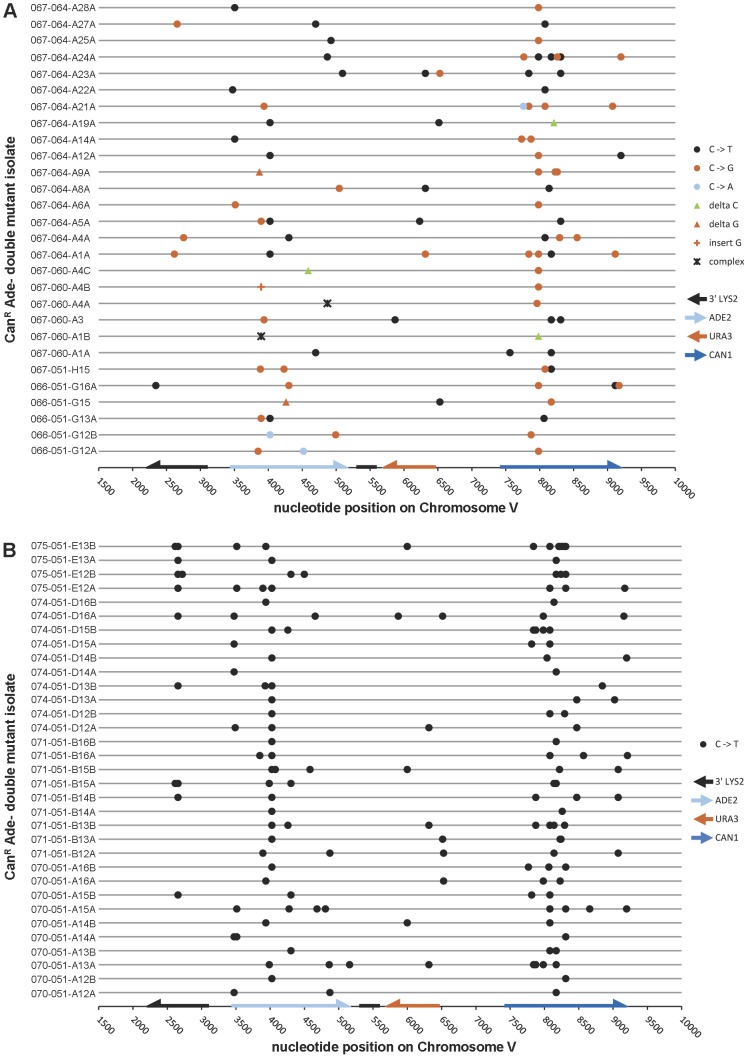
The mutation spectra of double loss-of-function isolates induced by APOBEC3G. (A) The reporter gene region of 28 Can^R^ Ade^−^ isolates obtained from *UNG1* cells expressing APOBEC3G was sequenced. 41.7% of mutations were C to T transitions, while 46.9% were C to G transversions. (B) Similarly, the reporter gene region of 33 Can^R^ Ade^−^ isolates obtained from *ung1Δ* cells expressing APOBEC3G was sequenced. All mutations were C to T transitions. Table S3 lists APOBEC3G-induced mutations, while Table S5 lists mutations found in empty vector controls.

To determine whether APOBEC3G expression actually resulted in a strand-coordinated, multi-mutation signature, we collected double loss-of-function isolates from populations of cells that expressed APOBEC3G during temperature shift and sequenced the three reporter genes, along with a ∼1 kb portion of *LYS2* that is telomere-proximal to *ADE2* in our reporter strains. Among 28 Can^R^ Ade^−^ isolates that were WT for *UNG1*, over 90% of mutations (86 out of 96) were base substitutions at cytosines on the ssDNA overhang strand (see Table 1 and Figure 3A). C to T transitions (41.7%) and C to G transversions (46.9%) occurred at similar frequencies. This is consistent with a mechanism where excision of uracil in the ssDNA by Ung1 [20] is followed by the action of a TLS polymerase to bypass the resulting abasic sites or to extend past the bypassed abasic sites [21].

**Table 1:** Compilation of APOBEC3G-induced mutations (sorted by type) and APOBEC3G motif preference.

	Mutation					Motif				
Genotype (no. of mutations)	C to T	C to G	C to A	complex	indel	CCC	TCC	ACC	GCC	other
***UNG1*** ** (96)**	41.7%	46.9%	3.1%	2.1%	6.3%	67.0%	19.1%	7.4%	1.1%	5.3%
***ung1Δ*** ** (150)**	100%	0%	0%	0%	0%	64.7%	24.7%	7.3%	1.3%	2.0%

Complex mutations are defined as two or more single base changes, where the 5′- and 3′-most changes are within ten nucleotides of one another. The 3′ C (underlined) in each motif triplet undergoes deamination. In rare cases, deamination occurred at an internal C within a run of >3 C's. Two complex mutations in *UNG1* were omitted from computation of motif preference. P<0.001 between *UNG1* and *ung1*Δ when comparing proportions of mutation types. P>0.05 when comparing proportions of motifs.

If Ung1 were solely responsible for excision of uracil formed by deamination of cytosine, there should be a significant enrichment for C to T transitions in *ung1*Δ cells expressing APOBEC3G concurrently with temperature shift. Consistent with this expectation, among 33 Can^R^ Ade^−^ isolates from the *ung1*Δ background, all 150 mutations in the reporter region were C to T transitions in the ssDNA overhang strand (see Table 1 and Figure 3B). For both WT and *ung1*Δ backgrounds, it was not uncommon to find reporters that harbored multiple mutations (up to 12 mutations, in isolate 075-051-E13B, see Table S3), reminiscent of the hypermutagenic action of APOBEC3G on minus strand pro-retroviral DNA [22].

Finally, we determined the motif specificity of APOBEC3G in the context of the subtelomeric reporter system. We found a strong preference for 5′-CCC-3′ motifs (where the underlined C is the mutated nucleotide), with a secondary preference for 5′-TCC-3′ motifs, while mutations at 5′-ACC-3′, and especially 5′-GCC-3′, were rare (see Table 1 and Figure S1). These observations are consistent with previous reports (e.g. [23]). We conclude that the ssDNA reporter system can readily detect mutagenesis induced by an enzyme that targets ssDNA. In addition, we have found that the action of an APOBEC3 cytosine deaminase indeed, can result in efficient generation of strand-coordinated, multi-mutation clusters within eukaryotic chromosomal DNA, in agreement with bioinformatics analysis of mutations in human cancers [5], [8].

### Acute treatment with sodium bisulfite induces localized hypermutability within subtelomeric ssDNA

Having validated our overall approach by using an enzymatic ssDNA-specific mutagen, we tested an agent of environmental relevance that might exhibit a similar mutational preference for ssDNA *in vivo*, namely sodium bisulfite (NaHSO_3_). In aqueous solution, the bisulfite anion (HSO_3_^−^) freely interconverts with sulfite anion (SO_3_^2−^) by deprotonation, and with hydrated sulfur dioxide (SO_2_•H_2_O) by protonation. In addition, two bisulfite anions can dehydrate to form a metabisulfite anion (S_2_O_5_^2−^) [24]. Since administration of one of these substances introduces the interconversion products as well, we refer collectively to all four sulfur (VI) oxides as “sulfites” hereafter. Previously, sulfites have been reported to induce genotoxicity in a number of model systems, although such effects were prone to poor reproducibility between different test systems and laboratories (see Discussion). The molecular mechanisms which could underlie such genotoxicity are not well understood. *In vitro* at high concentrations of ≥2 M (>20% by weight), sodium bisulfite deaminates cytosine (but not 5-methylcytosine) in denatured DNA to completion, which is the basis for the ‘bisulfite sequencing’ method to characterize DNA methylation [25].

**Figure 4:**
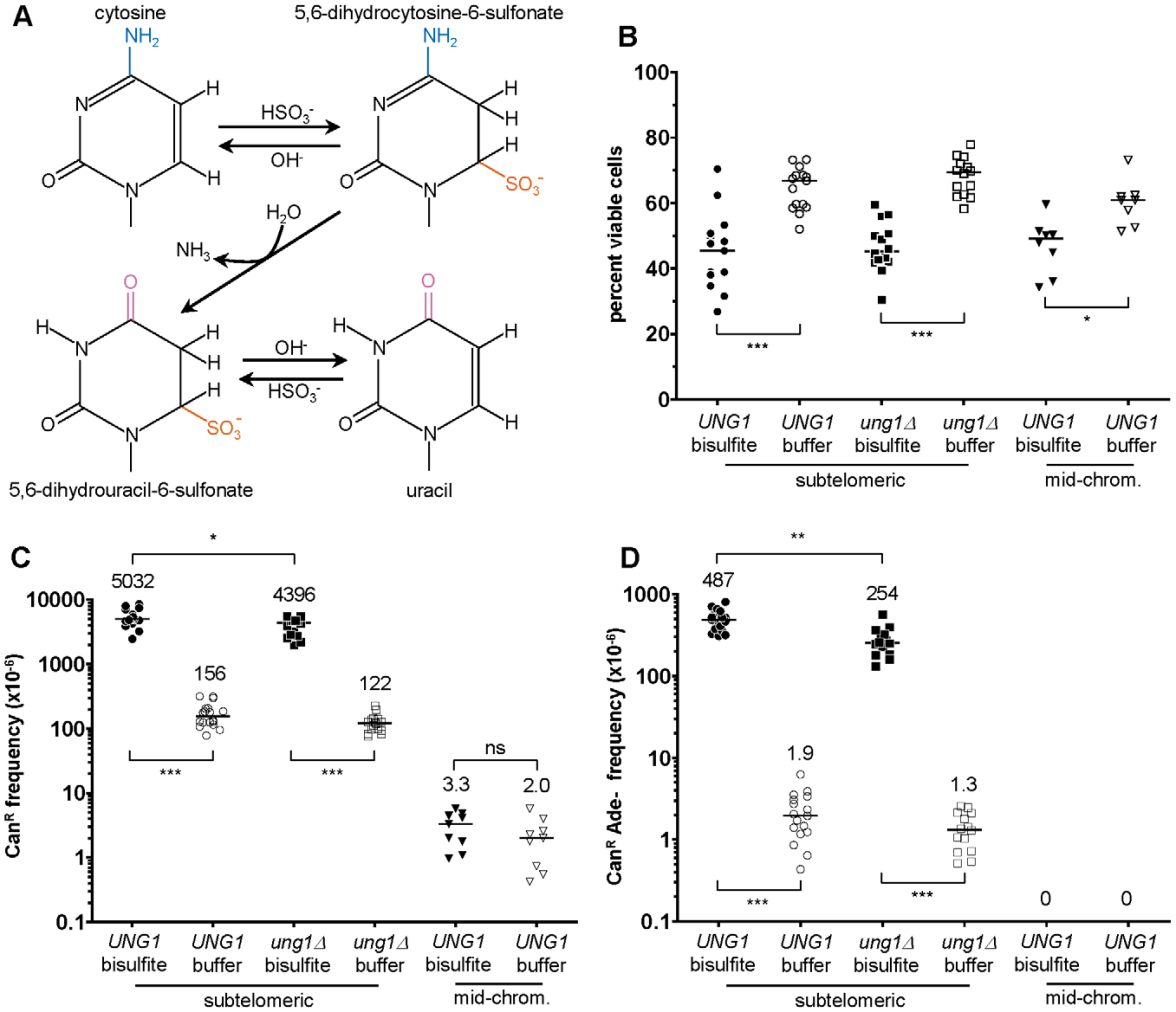
1% sodium bisulfite is a strong ssDNA–specific mutagen in vivo. (A) Chemical mechanism for bisulfite-mediated conversion of cytosine to uracil. Bisulfite anion reacts with cytosine at C6 to generate 5,6-dihydrocytosine-6-sulfonate, which in turn, undergoes irreversible hydrolytic deamination at C4 to generate 5,6-dihydrouracil-6-sulfonate. Finally, 5,6-dihydrouracil-6-sulfonate is desulfonated by reaction with hydroxide anion to form uracil. (B) Treatment with 1% sodium bisulfite resulted in a modest decrease in viability, with a median value of ∼45%. Buffer only controls exhibited a median viability of at least 60%. (C) 1% sodium bisulfite induced a 32- and 36-fold increase in the frequency of *CAN1* loss of function when ssDNA was formed, for *UNG1* and *ung1*Δ cells, respectively. Notice that there is no induced mutagenesis in mid-chromosome (i.e. dsDNA) controls. (D) 1% sodium bisulfite induced 250- and 195-fold increases in the frequency of simultaneous *CAN1* and *ADE2* double loss of function within ssDNA, for *UNG1* and *ung1*Δ cells, respectively.

Given these considerations, we tested whether a more moderate concentration of sodium bisulfite, similar to what would be expected in an environmental exposure to humans, might induce damage specifically in ssDNA *in vivo*, presumably by the chemical mechanism shown in Figure 4A [26]. After testing a range of different treatment conditions (e.g. see Figure S2), we chose 1% (∼100 mM) sodium bisulfite exposure for 2.5 hours, as this treatment resulted in only a modest (1.5-fold) decrease in viability (see Figure 4B) combined with very strong mutagenesis. Bisulfite induced a 32- and a 36-fold increase in the frequency of *CAN1* loss of function within subtelomeric ssDNA in WT and *ung1Δ* cells over buffer-only controls, respectively (P<0.001 in both cases, see Figure 4C). Similarly, bisulfite caused a 256- and a 195-fold increase in the frequency of simultaneous loss of both *CAN1* and *ADE2* function (P<0.001 in both cases, see Figure 4D). But in contrast to what we had observed with APOBEC3G, deletion of *UNG1* resulted in only a modest decrease in bisulfite-induced mutagenesis (compare Figure 2 to Figure 4).

### The spectrum of bisulfite-induced mutations confirms that the mechanism of mutagenesis is independent of *UNG1*

**Figure 5:**
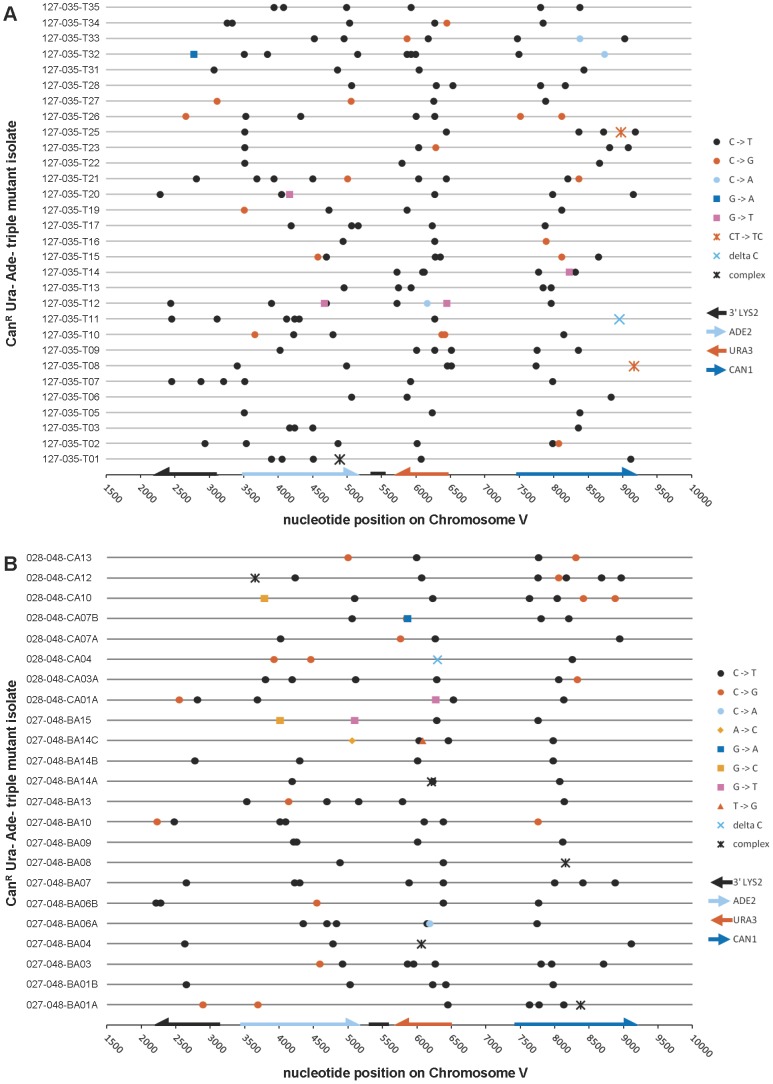
The mutation spectra of triple loss-of-function isolates induced by 1% sodium bisulfite. (A) The reporter gene region of 30 Can^R^ Ura^−^ Ade^−^ isolates obtained from bisulfite treatment of *UNG1* cells was sequenced. (B) The reporter gene region of 23 Can^R^ Ura^−^ Ade^−^ isolates obtained from bisulfite treatment of *ung1*Δ cells was sequenced. In both cases, >75% of mutations were C to T transitions, confirming that bisulfite-induced mutagenesis is independent of *UNG1* genotype. Table S4 lists bisulfite-induced mutations, while Table S6 lists mutations found in buffer only controls.

We next examined the spectrum of mutations appearing in Can^R^ Ura^−^ Ade^−^ triple loss of function mutants that resulted from exposure of subtelomeric ssDNA to bisulfite. We identified 166 mutations from 30 isolates that were wild-type for *UNG1*. 81.9% of these mutations were C to T transitions (see Table 2 and Figure 5A). C to G transversions comprised the second most common type of mutation, but only at a frequency of 10.8%. This was in marked contrast to what we had observed in WT cells expressing APOBEC3G, where the frequency of C to T transitions was roughly the same as that of C to G transversions (compare Table 1 to Table 2). Similarly, we found 124 mutations among 23 triple loss-of-function isolates from *ung1Δ* populations treated with bisulfite during temperature shift. 75.0% of these mutations were C to T transitions and 14.5% were C to G transversions (see Table 2 and Figure 5B). Taken together, these results suggest that the mechanism of mutagenesis induced by bisulfite is independent of uracil-DNA N-glycosylase, which in turn, suggests that the mutagenic lesion is not uracil.

**Table 2:** Compilation of bisulfite-induced mutations.

	Mutation					
Genotype (no. of mutations)	C to T	C to G	C to A	complex	indel	other
***UNG1*** ** (166)**	81.9%	10.8%	1.8%	0.6%	0.6%	4.2%
***ung1***Δ** (124)**	74.2%	14.5%	0.8%	4.0%	0.8%	5.6%

Complex mutations are defined as two or more single base changes, where the 5′- and 3′-most changes are within ten nucleotides of one another. P>0.05 between *UNG1* and *ung1*Δ when comparing the proportions of mutation types.

### Bisulfite-induced mutagenesis results from Pol ζ–dependent bypass of a long-lived sulfonated uracil intermediate

Given that Ung1 excised uracils formed by the action of APOBEC3G, but essentially had no effect on bisulfite-induced mutagenesis, we inferred that the reaction of bisulfite with cytosine resulted in a modified pyrimidine, distinct from uracil, which was recalcitrant to excision by Ung1. The two candidate modified pyrimidines are 5,6-dihydrocytosine-6-sulfonate and 5,6-dihydrouracil-6-sulfonate (see Figure 4A). Hayatsu and colleagues had shown previously that when bisulfite reacts with cytosine, the principle product obtained (at 72% yield) was 5,6-dihydrouracil-6-sulfonate [27]. They were unable to isolate the 5,6-dihydrocytosine-6-sulfonate intermediate in a stable form, apparently because it readily decomposes to re-form cytosine and bisulfite [27]. Thus, we consider sulfonated uracil as more likely to be a long-lived intermediate species in DNA within the bisulfite-treated cells.

**Figure 6:**
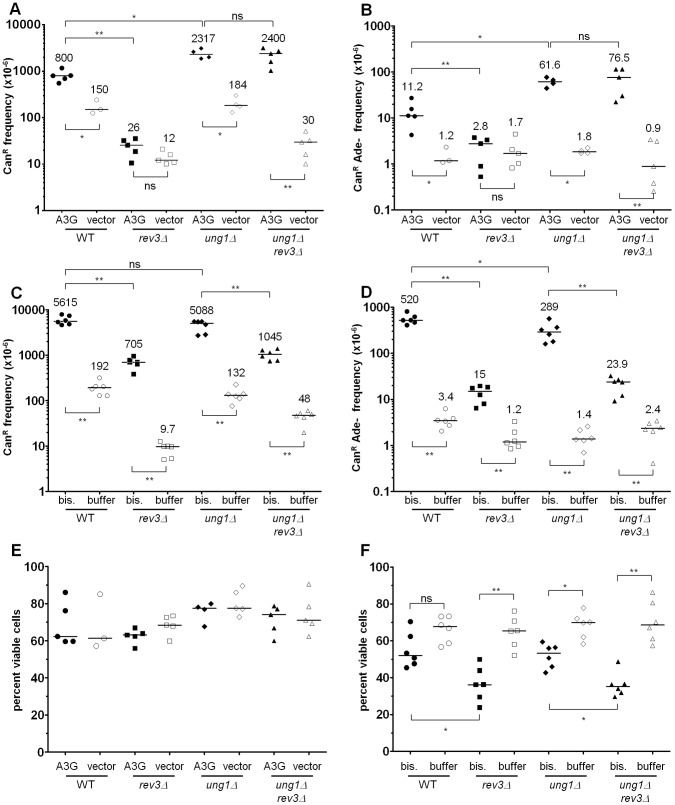
Pol ζ is involved in the majority of error-prone bypass of the 5,6-dihydrouracil-6-sulfonate intermediate of bisulfite-induced cytosine deamination. (A & B) The frequencies of Can^R^ (A) and Can^R^ Ade^−^ (B) in wild-type, *rev3*Δ, *ung1*Δ, and *ung1*Δ *rev3*Δ cells expressing APOBEC3G or bearing empty plasmids are shown. Notice that *REV3*, encoding the catalytic subunit of Pol ζ, is not required for mutagenesis in *ung1*Δ cells expressing APOBEC3G. (C & D) The frequencies of Can^R^ (C) and Can^R^ Ade^−^ (D) in wild-type, *rev3*Δ, *ung1*Δ, and *ung1*Δ *rev3*Δ cells that were treated either with bisulfite or with buffer only are shown. Comparison between *ung1*Δ and *ung1*Δ *rev3*Δ cells suggests that Pol ζ is required for the error-prone bypass of a bisulfite-induced lesion that is distinct from uracil. Taken together with previously published biochemical findings [27], [48], these results suggest that the 5,6-dihydrouracil-6-sulfonate intermediate exists in significant quantities *in vivo* as a consequence of the bisulfite reaction with cytosine. (E) APOBEC3G expression was well-tolerated in *rev3*Δ and *ung1*Δ *rev3*Δ cells. There was no statistically significant difference in viability when compared to WT or *ung1Δ* parental strains, respectively. (F) Bisulfite treatment of *rev3*Δ and *ung1*Δ *rev3*Δ cells resulted in statistically significant decreases in viability when compared to WT and *ung1Δ* parental strains, respectively. This indicates that decreased proficiency of translesion synthesis to bypass 5,6-dihydrouracil-6-sulfonate results in decreased viability.

If 5,6-dihydrouracil-6-sulfonate were the adducted uracil formed by bisulfite, then we hypothesized that a TLS polymerase could be required to perform mutagenic bypass during repair synthesis. Conveniently, the all-C-to-T mutation spectrum of *ung1*Δ cells expressing APOBEC3G suggested a suitable control experiment: TLS would be dispensable for mutagenesis when deaminated cytosines (i.e. uracils) are not excised from the ssDNA reporter, since a replicative polymerase should suffice to insert adenine opposite uracil. Consistent with this prediction, *UNG1* cells expressing APOBEC3G were dependent on *REV3* function for mutagenic bypass of abasic sites (*REV3* encodes the catalytic subunit for TLS polymerase ζ), but *ung1*Δ cells exhibited the same frequency of mutagenesis regardless of whether *REV3* was present (see Figure 6A and 6B). By contrast, bisulfite-induced mutagenesis was dependent on Pol ζ function, irrespective of *UNG1*. Deletion of *REV3* resulted in an 8- and a 5-fold decrease in the frequency of *CAN1* loss of function, in *UNG1* and *ung1*Δ cells, respectively (P<0.01 in both cases, see Figure 6C). Similarly, deletion of *REV3* resulted in a 35- and a 12-fold decrease in the frequency of simultaneous loss of both *CAN1* and *ADE2* function (P<0.01 in both cases, see Figure 6D). Thus, these results are consistent with bisulfite-induced formation of a relatively long-lived intermediate (5,6-dihydrouracil-6-sulfonate) that is excised inefficiently by Ung1. As a consequence of the persistence of 5,6-dihydrouracil-6-sulfonate in the subtelomeric ssDNA, a TLS polymerase (i.e. Pol ζ) is necessary to enable error-prone bypass when the DNA is restored to a double-stranded state, resulting in the observed strand-coordinated, multi-mutation signature.

Finally, we found that *rev3Δ* cells survived the bisulfite treatment less frequently than WT cells (P<0.05, see Figure 6F). Similarly, *ung1*Δ *rev3*Δ cells exhibited significantly lower survival than *ung1*Δ cells after bisulfite treatment (P<0.05, see Figure 6F). These data indicate that TLS-mediated bypass of 5,6-dihydrouracil-6-sulfonate is important for maintaining viability. In contrast, deletion of *REV3* in cells expressing APOBEC3G had no significant effect on survival (see Figure 6E), suggesting that cells possess alternative mechanisms for dealing with abasic sites in ssDNA, without compromising viability.

## Discussion

### A new tool to study mutagenesis

DNA in a single-strand state is more susceptible to many different forms of damage than the same DNA in double-strand form. Thus, there could be many agents that can damage ssDNA specifically *in vivo*, while being relatively inert toward dsDNA. The subtelomeric triple reporter gene system that we describe enables the facile identification and characterization of agents that induce lesions in ssDNA preferentially. Since the complementary strand of subtelomeric DNA is removed by exonucleolytic digestion after telomere uncapping, it is not possible for the cells to use excision repair to correct the lesions formed within the 3′ ssDNA overhang. Instead, cells are forced to use translesion synthesis bypass of the lesions, which if error-prone, generates a characteristic strand-coordinated, multi-mutation signature that is detected readily by plating on the appropriate selection media. The ssDNA is said to be locally hypermutable with respect to agents that induce such a mutation signature [5]–[7].

While the subtelomeric ssDNA reporter system identifies agents that *can* mutate ssDNA *in vivo*, additional studies are required to determine whether an agent in question *does* cause significant genome instability. Usually, ssDNA is formed only transiently during routine DNA transactions, which limits the time during which an agent in question could react with the ssDNA. In the case of base damage within ssDNA that arose from transient unwinding of duplex DNA, most of the lesions would be corrected by excision repair after the ssDNA re-anneals to form dsDNA, thus mitigating the mutagenic effects of the DNA damage. On the other hand, the prominent APOBEC mutagenic signature in some cancers [5], [8] would suggest that, at some point(s) during carcinogenesis, the ssDNA-specific APOBEC enzymes, and perhaps other ssDNA-damaging agents, do cause significant mutagenesis via transient ssDNA regions of the genome. In short, the impact of ssDNA-specific damage on overall genome stability is an open question that warrants much additional investigation.

### The fate of uracil formed by cytosine deamination in ssDNA

We have used the subtelomeric ssDNA reporter system to study mechanisms of mutagenesis by enzymatic and chemical cytosine deamination. APOBEC3G can induce deamination of multiple cytosines within the ssDNA overhang. The subsequent excision of uracils by the uracil-DNA N-glycosylase Ung1 was followed by error-prone bypass of the resulting abasic sites, which required Pol ζ, either for the actual bypass step or to extend from the base inserted opposite the abasic site [21]. This resulted in numerous simultaneous C to T and C to G mutations, all on the ssDNA overhang strand. We have considered also the possibility that the C to T transitions in WT cells expressing APOBEC3G could have resulted from the failure of Ung1 to excise a significant proportion of uracils from the ssDNA. But, this would predict that mutagenesis arising from the unexcised uracils would not require the participation of any TLS polymerase. If unexcised uracils were a significant contributor to mutagenesis, then overall frequency of mutagenesis in these cells should not be affected significantly by deletion of a TLS polymerase. Contrary to these predictions, mutagenesis in WT cells expressing APOBEC3G was decreased considerably (as much as 27-fold) when Pol ζ function was removed by deleting *REV3*. Thus, the presence of unexcised uracils in the ssDNA reporter is unlikely to be a major contributor to mutagenesis within WT cells.

Deletion of *UNG1* resulted in a mutation spectrum consisting entirely of C to T transitions on the ssDNA overhang strand. Interestingly, the overall mutation frequency was at least three-fold higher in *ung1x*Δ cells, suggesting that only a fraction of abasic sites created by Ung1 in WT cells ultimately led to mutations. In the absence of the DNA strand complementary to the ssDNA overhang, the abasic sites could not be repaired by BER. One possible explanation for the lower mutation frequency in WT cells is that the polymerase performing the actual bypass of abasic sites can insert a G with significant frequency. Alternatively, only a subset of abasic sites might be subject to TLS bypass. One potential mutation avoidance mechanism would involve bypass of the abasic site(s) during repair synthesis by template switching (see Figure S3). Alternatively, an abasic site could result in spontaneous strand breakage, or cause stalling of (replicative) DNA polymerase leading to strand breakage, followed by recombinational repair using the sister chromatid template (see Figure S4). This latter mechanism would be consistent with the reported recombinogenic effect caused by expression of the AID cytosine deaminase, observed in WT yeast, but not in *ung1*Δ [28].

In light of the confirmed capacity of Ung1 to excise uracils efficiently from ssDNA *in vivo*, further studies are needed to evaluate the relative contribution of mechanisms besides TLS toward the processing of abasic sites formed in ssDNA within different contexts. For instance, if abasic sites were formed in transiently unwound ssDNA, such as within transcription-bubbles, or R-loops that then re-anneal [4], error-free repair should be possible via BER. Similarly, error-free bypass of abasic sites generated in transient ssDNA of a replication fork could be mediated by a fork-reversal mechanism [29]. Intriguingly, uracil-DNA N-glycosylase (UNG2) activity has been co-localized with replication factories of mammalian cells [30]. Then it is possible that there is an additional mechanism to reduce the mutagenic effect of uracil in DNA, since uracil-DNA N-glycosylase would excise uracil encountered during genome replication, generating an abasic site that could be bypassed in an error-free manner by fork reversal.

### Sulfites are a class of environmental agents that can induce genotoxicity via poorly understood molecular mechanisms

The term ‘sulfites’ is used commonly to refer to several sulfur(IV) oxides that freely interconvert with one another in aqueous solution: sulfur dioxide (SO_2_), sulfite (SO_3_^2−^), bisulfite (HSO_3_^−^), and metabisulfite (S_2_O_5_^2−^). Over the decades, there have been many reports that sulfites induce genotoxicity. For instance, *Escherichia coli* and its phages, as well as *Salmonella typhimurium*, can tolerate large doses of bisulfite (1 M or ∼10% w/v, and above), and exhibit dose-dependent increases in mutation frequency [31]–[33]. Moreover, other groups reported that lower concentrations of bisulfite can induce weak mutagenesis, e.g. with 2 mM at pH 3.6 in *Salmonella* [34] and 100 mM at pH 5 and 6 in yeast [35]. In addition, there have been reports that sulfites induce chromosome aberrations, sister chromatid exchange, or micronuclei formation. Low doses induce these genotoxic outcomes in mammalian cells treated *in vitro* [36]–[39]. Substantially higher doses are required to observe similar abnormalities in cells isolated from rodents injected with sulfites [36], [40], [41]. Furthermore, bisulfite enhances UV-induced mutagenesis by two-fold in Chinese hamster cells and by eight-fold in *E. coli* [42]. Similarly, bisulfite enhances the mutagenicity of the activated form of benzo[a]pyrene in Chinese hamster cells by 2.5-fold [43].

Ironically, although there is considerable evidence that sulfites can induce genotoxicity, there are also many reports that sulfites are not genotoxic (see [44] and references therein). The reasons for such contradictory results from similar mutagenicity assays have not been clear. Our findings suggest a conceptual framework for reconsidering the ambiguous mutagenesis results previously reported for sulfites. At best, sulfites are relatively weak mutagens when tested using conventional reporter systems, where most of the time, DNA can be assumed to be in the canonical double-strand form. However, it has been shown that certain sequences in DNA are more prone to bisulfite-mediated cytosine deamination [45], because such sequences can exist in a non-B form state and the duplex is more likely to unwind partially, resulting in localized base pair opening [45], [46]. Thus, it is possible that in previously published mutagenesis reporter assays, slight differences in sulfite-induced mutability might be related to the sequence idiosyncrasies of the reporter DNA. All other factors being equal, duplex reporter DNA that is more prone to partial unwinding might be more likely to yield a positive assay result for sulfite-induced mutagenicity than reporter DNA that is not as prone to such unwinding.

### Bisulfite induces genotoxic long-lived 5,6-dihydrouracil-6-sulfonate ssDNA lesions *in vivo*

**Figure 7:**
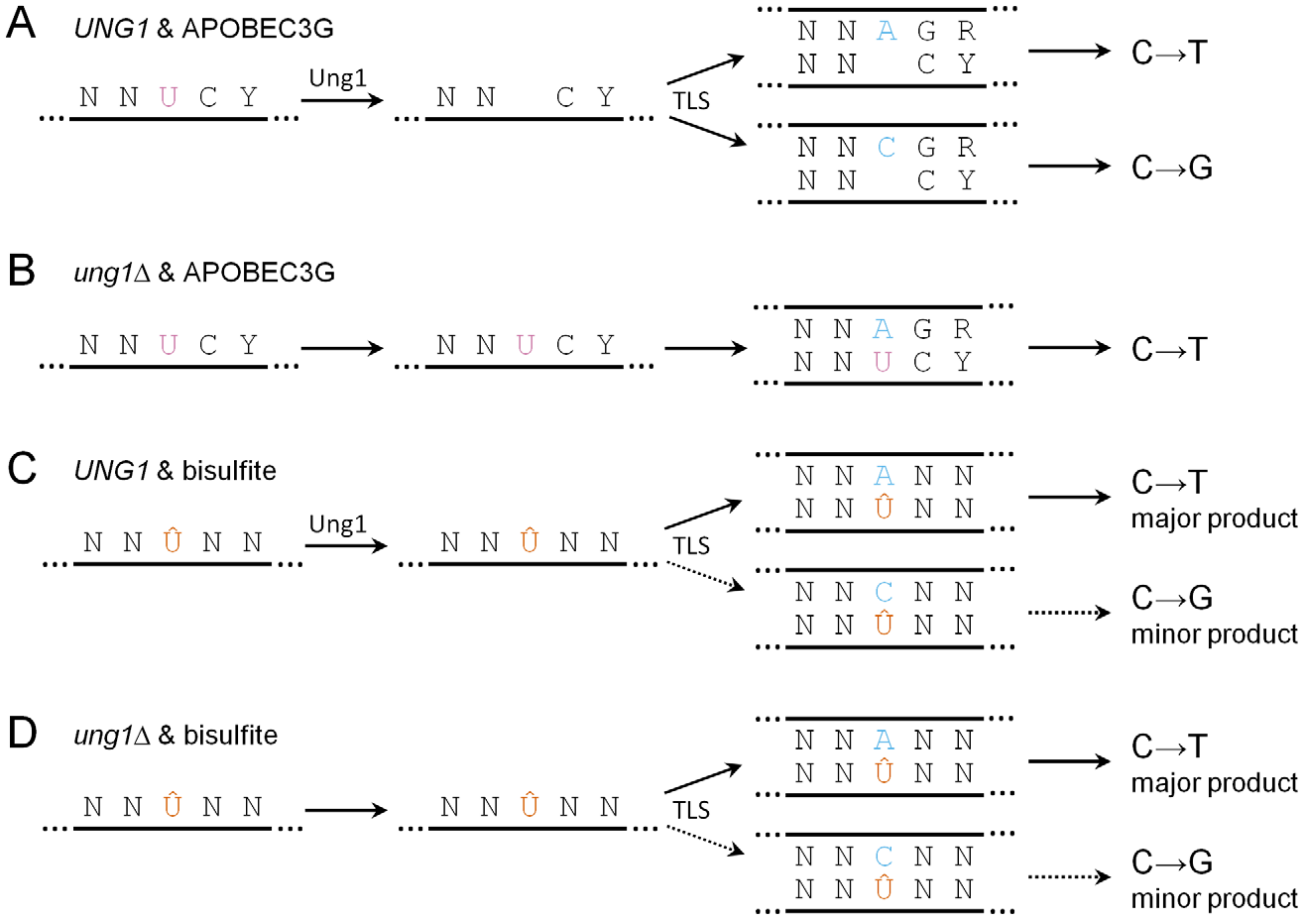
Summary of the possible fates of deaminated cytosine. Y denotes pyrimidine, R denotes purine, and N denotes any base. (A) In *UNG1* cells expressing APOBEC3G, uracil resulting from cytosine deamination (frequently at 5′-YCC-3′ motifs) is excised efficiently by Ung1. During repair synthesis, TLS inserts adenine or cytosine opposite abasic sites, resulting in similar frequencies of C→T transitions and C→G transversions. (B) In *ung1Δ* cells, uracil persists. During repair synthesis, adenine is inserted opposite uracil, resulting in all C→T transitions. (C) In *UNG1* cells treated with bisulfite, 5,6-dihydrouracil-6-sulfonate (denoted as Û) persists because of inefficient excision. During repair synthesis, TLS usually results in insertion of adenine opposite 5,6-dihydrouracil-6-sulfonate, while insertion of cytosine occurs infrequently. (D) In *ung1Δ* cells, 5,6-dihydrouracil-6-sulfonate also persists, resulting in similar mutagenic outcomes as *UNG1* cells.

While it has been appreciated that high concentrations of bisulfite induce cytosine deamination in ssDNA *in vitro* [25], it has not been possible to determine whether smaller concentrations, that are more relevant to levels of environmental exposure to humans, can induce cytosine deamination in ssDNA *in vivo*, since a suitable reporter system had not been available. Like APOBEC3G, we found that 1% bisulfite (which is less than three-fold greater than the highest concentration of sulfites found in commercial food products [10]) induces clusters of mutations at cytosines within subtelomeric ssDNA. But unlike APOBEC3G, bisulfite does not induce conversion of cytosine to uracil *per se*. Instead, bisulfite most likely generates an adducted uracil, namely 5,6-dihydrouracil-6-sulfonate. In contrast to uracil, replication past the sulfonated adduct often requires the translesion DNA polymerase Pol ζ (see Figure 6). The various possible fates of deaminated cytosine are summarized in Figure 7.

This sulfonate adduct to uracil could be particularly deleterious. First, it is relatively long-lived. The desulfonation step that converts this adduct to uracil is base-dependent (see Figure 4A). In neutral pH at 37°C, spontaneous desulfonation of the related adducted nucleoside occurs slowly: the half-life of 5,6-dihydrouridine-6-sulfonate is 66 minutes [47]. The half-life of the adducted nucleotide is likely to be even longer *in vivo* within the context of a DNA polymer, since the phosphate backbone would repel incoming OH^−^ anions electrostatically. In addition, consistent with previous biochemical results with the purified *E. coli* enzyme [48], we found evidence that the yeast uracil-DNA N-glycosylase Ung1 is inefficient at excising this adducted uracil *in vivo*. Indeed, bisulfite is a very potent mutagen of ssDNA, regardless of whether Ung1 is present in cells (see Figure 4C and 4D). Unlike the case with APOBEC3G, the presence of Ung1 did not affect the spectrum of mutations induced by bisulfite (compare Figure 3 to Figure 5). Instead, the mutation spectra for both *UNG1* and *ung1Δ* cells are mostly a reflection of mutagenic bypass enabled by Pol ζ, although it is possible that a small fraction of the adducted uracil underwent spontaneous desulfonation to form uracil.

In addition, we note that the 5,6-dihydrouracil-6-sulfonate lesion could be problematic even if formed within the context of transiently unwound ssDNA that re-anneals to re-form duplex DNA. Since 5,6-dihydrouracil-6-sulfonate probably is not subject to efficient BER initiated by Ung1, and the adduct likely would not be bulky enough to trigger nucleotide excision repair, it might not be possible to repair this lesion in a high fidelity manner, even within the context of re-annealed dsDNA. While spontaneous desulfonation could convert 5,6-dihydrouracil-6-sulfonate to uracil (which would be subject to BER), if the sulfonate adduct stays bound to uracil as the genome is being replicated, the cell could very well be forced to use error-prone TLS to synthesize past the lesion, potentially resulting in mutation.

### Toward elucidating novel patterns of mutagenesis *in vivo*

In a recent review [49], it was stated eloquently that, “The patterns of somatic mutation found in a cancer genome reflect the DNA damage and mutagenic processes that have been operative and the repair mechanisms that have mitigated their impact. Thus, the cancer genome can be likened to an archaeological record bearing the imprint of these processes.” Since a cancer genome reflects the superimposition of myriad influences acting on DNA, it can be difficult to identify the relevant mutagenic and repair processes unambiguously, due to an insufficient knowledge of the underlying molecular mechanisms. The work we describe here highlights a new approach to elucidate molecular mechanisms of mutagenesis associated with damage to ssDNA, which is a necessary complement to existing methods for studying mutagenesis.

We have found that regions of ssDNA can be much more mutable than dsDNA, yielding gene inactivation frequencies that can be greater by three orders of magnitude. Thus, it is possible that damage to regions of ssDNA could contribute to the acquisition of hundreds, or even thousands, of point mutations within a small number of cell generations during carcinogenesis. Indeed, Stratton and colleagues recently described the occurrence of dominant subclones of cells within mammary tumors. They proposed that these subclones originated from a quiescent phase where hundreds to thousands of point mutations were accumulated with minimal proliferation, followed by clonal expansion to become the predominant cell lineage within a tumor [50]. Since a significant fraction of mutations in these tumors occurred at motifs that are characteristic of APOBEC enzymes [8], a more complete understanding of the mechanisms that contribute to ssDNA damage-driven mutagenesis could shed light on the molecular events that lead to the emergence of such subclones during tumorigenesis.

Finally, we point out that although there were clusters of multiple strand-coordinated mutations in cancer that are attributable to the action of ssDNA-specific APOBEC cytosine deaminases, such APOBEC-associated clusters comprised only 30% of all mutation clusters (120 out of 394) [5]. Clearly, other unidentified mutagenic processes were at work to generate mutation clusters, perhaps by acting on ssDNA intermediates. Further studies could reveal the identity of additional agents that are capable of inducing localized hypermutability within ssDNA, which in turn, could help to decipher the complex archaeological record of human cancer genomes.

## Materials and Methods

### Yeast strains

All yeast strains used in this study are isogenic to CG379 [51] with the following common markers: *MATα his7-2 leu2-3,112 trp1-289*. The *ADE2*, *CAN1*, and *URA3* genes were deleted from their native locations and reintroduced into the left subtelomeric region of Chromosome V using the *delitto perfetto* approach [52]. The *cdc13-1* mutation was introduced using the integration/pop-out technique described in [11]. Introduction of *cdc13-1* was verified by phenotype, restriction digest, and sequencing. *ung1*Δ and *rev3x*Δ derivative strains were constructed by one-step gene replacement [53] using the kanMX4 and natMX4 [54] antibiotic resistance markers, respectively. Deletion strains were confirmed by phenotype and PCR. Yeast strains were grown on YPDA liquid (1% yeast extract, 2% peptone, 2% dextrose, supplemented with 0.01% adenine sulfate, filter-sterilized) or agar (same recipe as YPDA liquid with 2% agar added, autoclaved), except for strains bearing tetracycline regulatable plasmids, which were maintained on TRP dropout plates.

### Plasmids

The tetracycline regulatable centromeric shuttle plasmid pCM252 [18] was a gift from Prof. E. Herrero (Universidad de Lleida, Spain). The APOBEC3G198-384-2K3A ORF [16], [17] was amplified from plasmid pGST-A3G-CTD-2K3A [55] by PCR using primers that introduced ClaI and StuI restriction sites. The APOBEC3G fragment then was cloned into pCM252 to generate pCM252-A3G. Correctly cloned plasmid isolates were identified by sequencing of the APOBEC3G ORF, as well as flanking sequences extending approximately 200 bp in the 5′ and 3′ directions. Plasmids were transformed into yeast using a standard lithium acetate technique [52].

### APOBEC3G-induced mutagenesis

Reporter strains bearing pCM252-A3G were maintained on TRP dropout plates. Individual colonies were inoculated each into a 5-mL YPDA liquid culture and grown at 23°C for 48 hours. 0.5 mL of each 48-hour culture was combined with 4.5 mL of fresh YPDA supplemented by 10 µg/mL doxycycline (Sigma-Aldrich, St. Louis, MO), and shifted to 37°C for 6 hours. Then, cells were collected by centrifugation, washed twice in water, and appropriate dilutions were plated onto ARG dropout plates with 60 µg/mL canavanine sulfate and 20 µg/mL adenine sulfate to select for mutants, as well as onto synthetic complete plates to assess viability.

### Bisulfite-induced mutagenesis

Individual colonies from each reporter strain each were inoculated into a 5-mL YPDA culture, and grown at 23°C for 72 hours. 0.5 mL of each 72-hour culture was combined with 4.5 mL of fresh YPDA, and shifted to 37°C for 6 hours. Then, cells were collected, washed twice, and counted. 2×10^7^ cells from each culture were resuspended in 1 mL of 1% sodium bisulfite in 100 mM sodium citrate buffer, pH 5.2, and incubated at 37°C for 2.5 hours. Then, cells were collected by centrifugation, washed twice in water, and plated as described for APOBEC3G-induced mutagenesis.

### Characterization of multi-loss-of-function strains

Plates with mutagenized cells were incubated at 23°C for five days, and counted using an aColyte 7510/SYN colony counter (Microbiology International, Frederick, MD). Replicas of selection media plates were made onto ADE dropout, URA dropout, and glycerol plates to identify multi-loss-of-function isolates that retained mitochondrial function. Isolates of interest were streaked onto YPDA and an individual colony from each streaking was tested to verify loss of function in the reporter genes. Genomic DNA was prepared by a QIAcube robot (QIAGEN, Valencia, CA), using the manufacturer's protocol. Each reporter ORF, as well as the 3′ portion of *LYS2*, was amplified by PCR using the primers listed in Table S1. Sequencing of these PCR products was outsourced to Eton Biosciences (Research Triangle Park, NC) using the primers listed in Table S2. Mutations were identified using Seqman software (DNASTAR, Madison, WI) and graphed using Excel (Microsoft, Redmond, WA).

### Statistical analyses

Prism 6 software (GraphPad Software, LaJolla, CA) was used to evaluate statistical significance of the data. The Kolmogorov-Smirnov test was applied to evaluate statistical significance of differences in viability and frequency of gene inactivation (i.e., data in Figure 2, Figure 4, and Figure 6). The Chi-square test was used to compare the difference in proportions of mutation types or motif preference between WT and *ung1*Δ cells (i.e., the data in Table 1 and Table 2).

## Supporting Information

Figure S1 Motif preference of APOBEC3G in subtelomeric ssDNA. The motif preference of APOBEC3G acting on subtelomeric ssDNA in (A) *UNG1* and (B) *ung1Δ* cells is shown. Primary preference is for 5′-CCC-3′ triplets, with a secondary preference for 5′-TCC-3′. Deamination occurs at the 3′ C of each triplet. In rare cases, deamination occurred at an internal C within a run of >3 C's.
(TIF)

Figure S2 The effect of reducing bisulfite exposure on mutagenesis. (A) Reducing duration of bisulfite exposure by 50% (i.e. 1% for 75 minutes) decreased the frequency of *CAN1* loss of function by 43%. Similarly, reducing the dose of bisulfite by 50% (0.5% for 150 minutes) decreased the frequency of *CAN1* loss of function by 38%. (B) 1% for 75 minutes resulted in a 64% decrease in the frequency of simultaneous loss of *CAN1* and *ADE2* function, while 0.5% for 150 minutes resulted in a 68% decrease, compared to 1% for 150 minutes.
(TIF)

Figure S3 Proposed mechanism for mutation avoidance by template switching. (A) A resected chromatid with an abasic site in the 3′ ssDNA overhang is depicted in orange, while the intact sister chromatid is shown in blue. (B) Repair synthesis stalls at the abasic site. (C) The newly synthesized segment of the orange top strand then strand invades the blue duplex. Extension results, essentially, in an error-free bypass of the abasic site. (D) The newly extended orange top strand re-anneals to the orange bottom strand. DNA synthesis resumes. (E) Completion of gap filling synthesis restores duplex DNA. (F) The newly synthesized top strand serves as a template for error-free base excision repair of the abasic site.
(TIF)

Figure S4 Proposed mechanism for mutation avoidance by breakage-associated homology-directed repair. (A) A resected chromatid with an abasic site in the 3′ ssDNA overhang is depicted in orange, while the intact sister chromatid is shown in blue. (B) A strand break occurs at a position 5′ of the abasic site. (C) The truncated 3′ overhang strand invades the intact blue sister chromatid. (D) Synthesis to the end of the chromosome restores the 3′ overhang to full length in an error-free manner. (E) Synthesis of the top orange strand restores duplex DNA.
(TIF)

Table S1 Primers used to amplify subtelomeric reporter gene region.
(DOCX)

Table S2 Primers used to sequence subtelomeric reporter gene region.
(DOCX)

Table S3 Catalog of mutations induced by APOBEC3G in subtelomeric ssDNA.
(XLSX)

Table S4 Catalog of mutations induced by bisulfite in subtelomeric ssDNA.
(XLSX)

Table S5 Catalog of mutations found in *CAN1* among temperature-shifted cdc13-1 cells bearing pCM252 with no insert.
(XLSX)

Table S6 Catalog of mutations found in *CAN1* among temperature-shifted *cdc13-1* cells treated with 100 mM citrate buffer, pH 5.2.
(XLSX)
