# Supplementary figures and images for: Base Damage within Single-Strand DNA Underlies In Vivo Hypermutability Induced by a Ubiquitous Environmental Agent
Source: PLoS Genet. 2012 Dec 13;8(12):e1003149. doi: 10.1371/journal.pgen.1003149 (PMC3521656; doi:10.1371/journal.pgen.1003149)

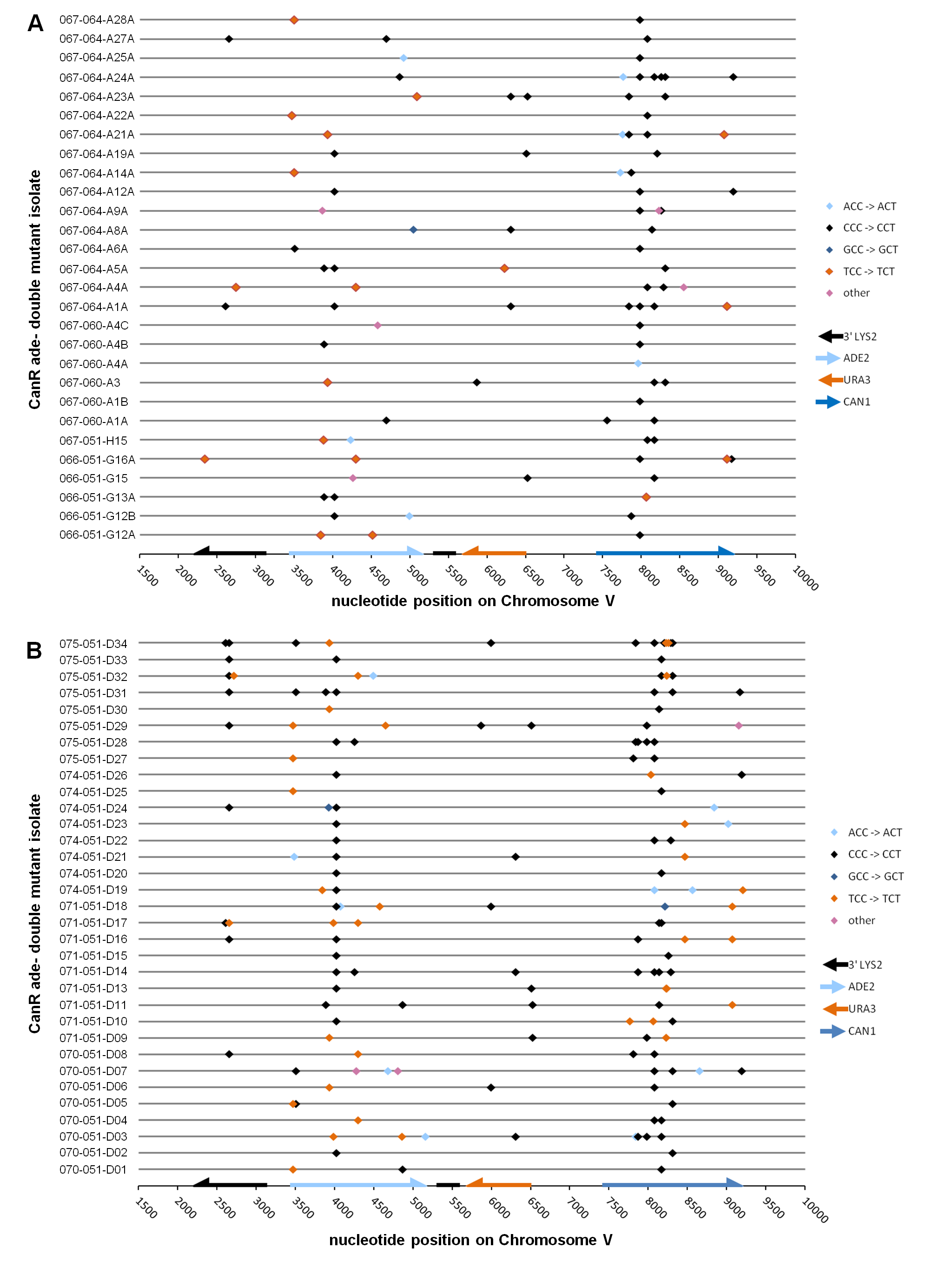

Supplement: Figure S1 — Motif preference of APOBEC3G in subtelomeric ssDNA. The motif preference of APOBEC3G acting on subtelomeric ssDNA in (A) UNG1 and (B) ung1Δ cells is shown. Primary preference is for 5′-CCC-3′ triplets, with a secondary preference for 5′-TCC-3′. Deamination occurs at the 3′ C of each triplet. In rare cases, deamination occurred at an internal C within a run of >3 C's. (TIF) [file pgen.1003149.s001.tif]

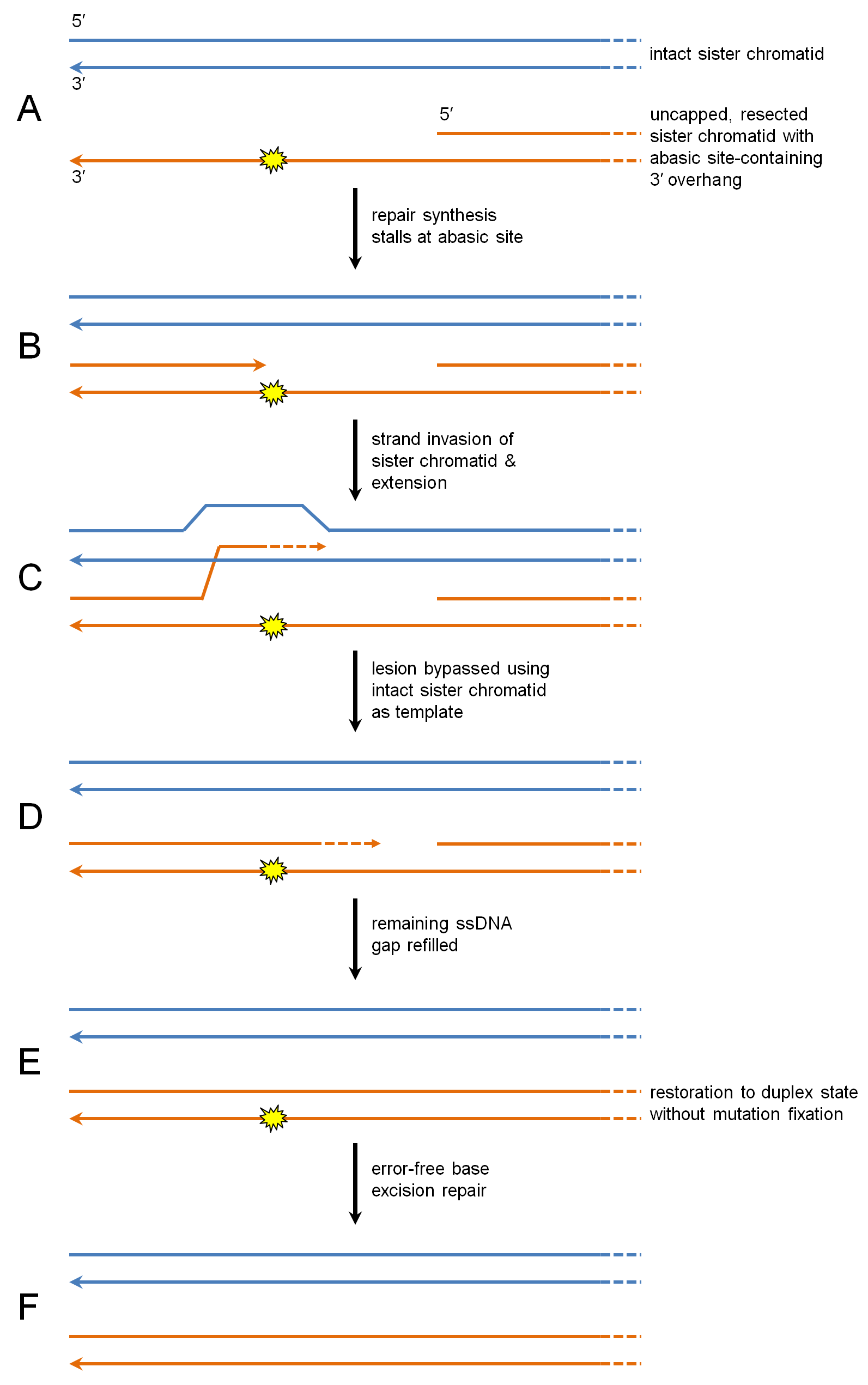

Supplement: Figure S3 — Proposed mechanism for mutation avoidance by template switching. (A) A resected chromatid with an abasic site in the 3′ ssDNA overhang is depicted in orange, while the intact sister chromatid is shown in blue. (B) Repair synthesis stalls at the abasic site. (C) The newly synthesized segment of the orange top strand then strand invades the blue duplex. Extension results, essentially, in an error-free bypass of the abasic site. (D) The newly extended orange top strand re-anneals to the orange bottom strand. DNA synthesis resumes. (E) Completion of gap filling synthesis restores duplex DNA. (F) The newly synthesized top strand serves as a template for error-free base excision repair of the abasic site. (TIF) [file pgen.1003149.s003.tif]

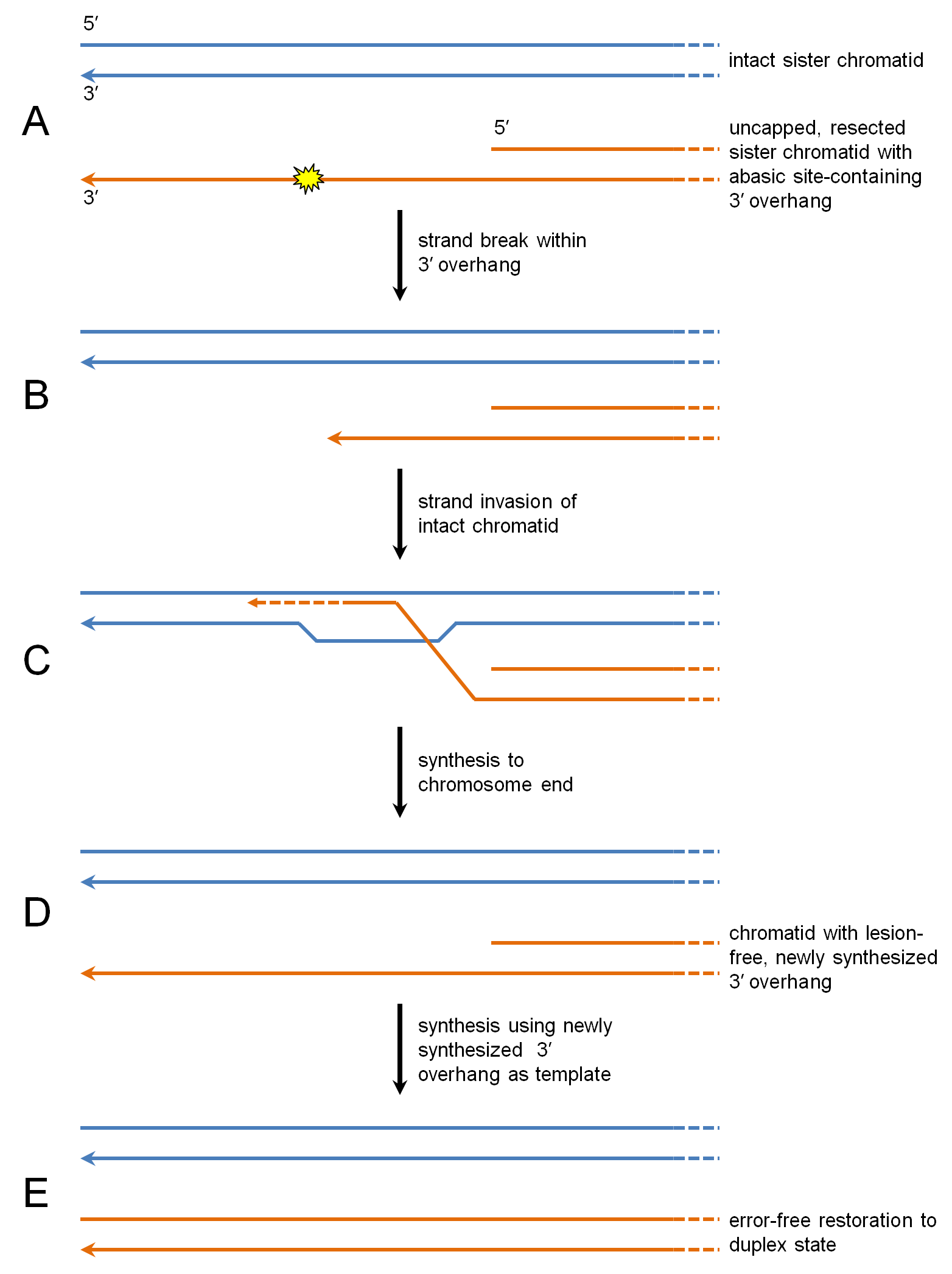

Supplement: Figure S4 — Proposed mechanism for mutation avoidance by breakage-associated homology-directed repair. (A) A resected chromatid with an abasic site in the 3′ ssDNA overhang is depicted in orange, while the intact sister chromatid is shown in blue. (B) A strand break occurs at a position 5′ of the abasic site. (C) The truncated 3′ overhang strand invades the intact blue sister chromatid. (D) Synthesis to the end of the chromosome restores the 3′ overhang to full length in an error-free manner. (E) Synthesis of the top orange strand restores duplex DNA. (TIF) [file pgen.1003149.s004.tif]
