# Supplementary material for: Base Damage within Single-Strand DNA Underlies In Vivo Hypermutability Induced by a Ubiquitous Environmental Agent
Source: PLoS Genet. 2012 Dec 13;8(12):e1003149. doi: 10.1371/journal.pgen.1003149 (PMC3521656; doi:10.1371/journal.pgen.1003149)
Supplement: Table S1 — Primers used to amplify subtelomeric reporter gene region. (DOCX) [file pgen.1003149.s005.docx]

| Primer Name | Sequence | Purpose |
| --- | --- | --- |
| oKC109 | TTAAGCTGCTGCGGAGCT | PCR of 3′ portion of *LYS2* |
| oKC110 | AGCCATGCAACAAGAGTC | PCR of 3′ portion of *LYS2* |
| oKC073 | CGAACCGGGTAATACTAAGTG | PCR of *ADE2* |
| oKC074 | GCCAAATTGAGGGATCTTATG | PCR of *ADE2* |
| oKC089 | GGTATTTCACACCGCATAG | PCR of *URA3* |
| oKC090 | GGTAATCTCCGAACAGAAG | PCR of *URA3* |
| oKC142 | GGTTGCGAACAGAGTAAACC | PCR of *CAN1* |
| oKC062 | AGGGTGAGAATGCGAAATG | PCR of *CAN1* |

Table S1. Primers used to amplify subtelomeric reporter gene region.
