# Supplementary material for: Base Damage within Single-Strand DNA Underlies In Vivo Hypermutability Induced by a Ubiquitous Environmental Agent
Source: PLoS Genet. 2012 Dec 13;8(12):e1003149. doi: 10.1371/journal.pgen.1003149 (PMC3521656; doi:10.1371/journal.pgen.1003149)
Supplement: Table S2 — Primers used to sequence subtelomeric reporter gene region. (DOCX) [file pgen.1003149.s006.docx]

| Primer Name | Sequence | Purpose |
| --- | --- | --- |
| oKC109 | TTAAGCTGCTGCGGAGCT | sequencing 3′ portion of *LYS2* |
| oKC110 | AGCCATGCAACAAGAGTC | sequencing 3′ portion of *LYS2* |
| seqDG_94 | AGGATACAACGCATTTTC | sequencing 3′ portion of *LYS2* |
| seqDG_99 | CGAAAATTCCGTGAATAT | sequencing 3′ portion of *LYS2* |
| oKC073 | CGAACCGGGTAATACTAAGTG | sequencing *ADE2* |
| oKC074 | GCCAAATTGAGGGATCTTATG | sequencing *ADE2* |
| seqKC002 | AGGACTTTGGCATACGATGG | sequencing *ADE2* |
| seqKC004 | ACTCTGACTTGCCGGTAATG | sequencing *ADE2* |
| seqKC007 | GTTCACATTCCGCCATACTG | sequencing *ADE2* |
| seqKC008 | AGTGACGCAAGCATCAATGG | sequencing *ADE2* |
| oKC089 | GGTATTTCACACCGCATAG | sequencing *URA3* |
| oKC090 | GGTAATCTCCGAACAGAAG | sequencing *URA3* |
| seqDG_78 | CTCCAGTAGATAGGGAGC | sequencing *URA3* |
| seqDG_81 | CGGGTGTATACAGAATAG | sequencing *URA3* |
| oKC062 | AGGGTGAGAATGCGAAATG | sequencing *CAN1* |
| seqDG_83 | CAAATTCAAAAGAAGACG | sequencing *CAN1* |
| seqDG_84 | ACGCAGTCCTTGGGTGAA | sequencing *CAN1* |
| seqDG_86 | TTGGTCTATCAAAGAACA | sequencing *CAN1* |
| seqDG_89 | AACTCGTCACGAGAGATG | sequencing *CAN1* |
| seqDG_91 | TTTGACAGGGAACAAGTT | sequencing *CAN1* |

Table S2. Primers used to sequence subtelomeric reporter gene region.
